# T-bet and interleukin-27: possible T_H_1 immunomodulators of sarcoidosis

**DOI:** 10.1007/s10787-015-0247-y

**Published:** 2015-08-09

**Authors:** Wei Sheng Joshua Loke, Araluen Freeman, Linda Garthwaite, Silvie Prazakova, Mijeong Park, Kenneth Hsu, Paul S. Thomas, Cristan Herbert

**Affiliations:** School of Medical Sciences, Inflammation and Infection Research Centre, UNSW, Sydney, 2052 Australia; Department of Respiratory Medicine, Prince of Wales Hospital, Randwick, NSW 2031 Australia

**Keywords:** Sarcoidosis, Anergy, T_H_1, Interferon-γ, T-bet, IL-27

## Abstract

**Background:**

Sarcoidosis has often been termed an “immune paradox” as there is peripheral anergy to common recall antigens despite pronounced T_H_1-dominant inflammation at disease sites, such as the lung, with up-regulation of interferon γ, IL-27 and transcription factors. Peripheral blood may reflect the anergic state, while exhaled breath condensate (EBC) analysis may offer insights into the lung disease.

**Methods:**

A cross-sectional study was conducted to investigate the expression of T_H_1 cytokines and transcription factors (IFNγ, IL-27 and T-bet) in the peripheral blood and/or EBC of sarcoidosis patients and healthy controls. Whole blood and EBC were collected from sarcoidosis patients and healthy controls. T_H_1 cytokine expression levels were then measured in peripheral blood mononuclear cells (PBMCs) and/or plasma and EBC using quantitative real-time PCR, ELISA and via Western blotting.

**Results:**

Compared to healthy controls, PBMC IL-27 mRNA was higher in patients (*p* = 0.0019). There were no significant differences in plasma IL-27 protein between patients and controls (*p* = 0.20). T-bet mRNA and protein were lower (*p* = 0.010 and *p* = 0.0043, respectively) in patients compared to controls. There were no significant differences in PBMC IFNγ mRNA and protein expression (*p* = 0.68 and *p* = 0.74, respectively) nor in EBC IL-27 levels.

**Conclusions:**

Our data indicate that depressed T-bet mRNA and protein expression could contribute to the peripheral anergy in sarcoidosis and that IL-27 mRNA levels are elevated in the PBMC from those with sarcoidosis.

**Electronic supplementary material:**

The online version of this article (doi:10.1007/s10787-015-0247-y) contains supplementary material, which is available to authorised users.

## Introduction

Sarcoidosis is a multi-system granulomatous disorder of undetermined aetiology (Loke et al. 2013). Lymphocytes in sarcoid granulomas are strongly T_H_1 polarised and are characterised by an amplified T-bet expression and IFNγ production (Gharaee-Kermani et al. 2009). Sarcoidosis is also often termed an “immune paradox” as there is peripheral anergy shown by a diminished delayed-type hypersensitivity to common recall antigens in the skin despite exaggerated inflammation at disease sites (Miyara et al. 2006; Morgenthau and Iannuzzi 2011). It has been suggested that underpinning this phenomenon is a disequilibrium between effector and regulatory lymphocytes (T_reg_ cells), more specifically CD4^+^CD25^bright^FoxP3^+^ cells (Mathew et al. 2008), which congregate in the peripheral blood and exercise anti-proliferative effects on naïve T-cells. Others suggest that the exaggerated immune response at disease sites results in the accumulation of activated T-cells at these sites and consequently, peripheral lymphopenia (Hunninghake et al. 1979). It is also possible that in chronic sarcoidosis, immunosuppressive CD8^+^ T-cells become more plentiful peripherally, thus effecting an anergic response (Planck et al. 2003). Despite intensive research upon pro-inflammatory mechanisms, there is a paucity of studies investigating the roles of immunosuppressive cytokines in the regulation of inflammation in sarcoidosis.

IL-27 is a heterodimeric cytokine structurally related to IL-12 (Villarino et al. 2004). It has recently been increasingly labelled as an attenuator of inflammatory responses. Mice that have been depleted of the IL-27 receptor WSX-1 produced more pro-inflammatory cytokines when infected with *Trypanosoma cruzi* and *T. gondii* (Villarino et al. 2003). IL-27 was also shown to repress cytokine production from activated T lymphocytes in vitro (Yoshimura et al. 2006) and promote IL-10 production (Stumhofer et al. 2007). Other mechanisms include inhibiting the differentiation of T_H_17 cells (Stumhofer et al. 2006) and activating the programmed cell death proteins, PD-1–PD-L1 (Topalian et al. 2012). Although Larousserie et al. (2004) have demonstrated the presence of Epstein-Barr virus-induced gene 3 and p28 (subunits of IL-27) in sarcoidosis lymph node biopsies using immunohistochemistry, there are, to date, no studies quantifying the expression of IL-27 in sarcoidosis.

Sarcoidosis remains a diagnosis of exclusion best confirmed by histological evidence showing non-caseating granulomas in the absence of granulomatous agents (Baughman et al. 2011). This necessitates a tissue biopsy which is invasive. Exhaled breath condensate (EBC) offers a non-invasive approach to sampling the fluid lining the respiratory tract and (Kazani and Israel 2012) our group has detected significantly elevated markers of macrophage activity (i.e. TNF-α, TGF-β_1_ and neopterin) (Ahmadzai et al. 2013) and IFNγ (Huang et al. 2013) in sarcoidosis EBC compared to healthy control subjects. There are, to our knowledge, no studies on IL-27 in peripheral blood mononuclear cells (PBMCs) and EBC of sarcoidosis patients.

In sarcoidosis, the expressions of IFNγ and Tbx21 in CD4^+^ T-cells are up-regulated post-T-cell activation. Together with STAT-4, interleukin-12 (IL-12) up-regulates IFNγ expression. IFNγ then stimulates STAT-1 which intensifies the expression of T-bet by the Tbx21 gene. T-bet enhances the transcriptional competence of the IFNγ gene and increases IFNγ production. T-bet also antagonises Gata3, therefore, down-regulating T_H_2 cytokines in the acute phase of sarcoidosis. Given that T-bet consigns T cells into the T_H_1 subset and the ability of IL-27 to mitigate inflammatory responses, we studied the expression of the above-mentioned T_H_1 modulators in the peripheral blood of sarcoidosis patients with the goal of elucidating whether they were associated with sarcoidosis peripheral anergy.

## Methods

### Study design

This was an observational, cross-sectional study which was approved by the Human Research Ethics Committees of St. Vincent’s Hospital and Prince of Wales Hospital, Sydney Australia (ethics approval number: 10/005). Informed, written consent was obtained from all participants. Sarcoidosis patients visiting the Prince of Wales Hospital Chest and Sarcoidosis Clinics and healthy controls from both the community and Prince of Wales Hospital were invited to participate. Patient inclusion criteria necessitated a diagnosis of sarcoidosis established prior to participation, based on the guidelines from the World Association of Sarcoidosis and Other Granulomatous Diseases (WASOG), the American Thoracic Society and the European Respiratory Society (Hunninghake et al. 1999). Patients on immunosuppressive medication were not excluded. Control subject criteria required a FEV_1_/FVC ratio of >80 % on spirometry and no significant medical history of atopic, granulomatous or autoimmune disorders.

### EBC collection and storage

EBC samples were collected using the EcoScreen condenser (Erich Jaeger GmbH, Hochberg, Germany). Subjects were asked to breathe for 15 min (tidal breathing) into a mouthpiece attached to a one-way valve. The EBC samples were stored in Protein LoBind tubes (Eppendorf, Sydney, Australia) in 120 µl aliquots, de-aerated with argon gas to remove CO_2_ and stored at −80 °C until analysis.

### Blood processing: plasma and PBMC isolation and cryopreservation

Approximately, 50 ml of blood was obtained in acid citrate dextrose-containing Vacutainer tubes (BD Biosciences, Sydney, Australia). Whole blood was centrifuged at 700×*g* for 10 min. The supernatant, plasma (10 ml), was removed, separated into 1 ml aliquots and stored at −80 °C until further analysis. PBMCs were then isolated by density gradient centrifugation using Ficoll-Hypaque Lymphoprep (STEMCELL Technologies, Melbourne, Australia) (Böyum 1968) and were kept in vapour-phase nitrogen at −190 °C until further analysis.

### RNA extraction from PBMC and EBC

Prior to cryopreservation, 5 million PBMCs were separated, lysed with TRIzol (Life Technologies, Melbourne, Australia) and stored at −80 °C until total RNA extraction according to the manufacturer’s protocol. The extracted RNA was resuspended in 20 µl of diethylpyrocarbonate-treated water. RNA concentration and purity were assessed using the NanoDrop 1000 Spectrophotometer (Thermo Fisher Scientific, Melbourne, Australia).

### cDNA synthesis and qRT-PCR analysis

RNA (≥1.0 µg) from PBMC was used for first-strand cDNA synthesis using a Superscript III first-strand synthesis kit (Invitrogen, CA, USA), following the manufacturer’s protocols. Quantitative real-time PCR for mRNA detection was performed using Sensimix SYBR (Bioline, London, UK) on the Light Cycler 480 (Roche, Mannheim, Germany) using standard conditions with hypoxanthine–guanine phosphoribosyltransferase (HPRT) as the reference control.

### Primers

Custom primers were designed using Primer3 (http://primer3.ut.ee/) and were purchased from either Invitrogen or Integrated DNA technologies (Singapore) (see Table 1 for primer sequences).

**Table 1:** Primer sequences for quantitative real-time PCR

Gene name	Primer sequences (5′–3′)		Product length (bp)
IFNγ human	F	TCGGTAACTGACTTGAATGTCCA	100
	R	TCCTTTTTCGCTTCCCTGTTTT	
IL-27 human	F	GGAATCTCACCTGCCAGGAGTG	128
	R	TGGTGGAGATGAAGCAGAGACG	
T-bet human	F	GATGCGCCAGGAAGTTTCA	142
	R	CTCTCCGTCGTTCACCTCAC	
HPRT human	F	GAAGAGCTATTGTAATGACC	177
	R	GCGACCTTGACCATCTTTG	

### Protein extraction from PBMC

Cryovials containing PBMCs were thawed in a 37 °C water bath. PBMCs were centrifuged at 400×*g* for 10 min at 4 °C and washed twice with DPBS. The supernatant was discarded and total protein was extracted using cell lysis buffer (Cell Signalling Technology, MA, USA) containing phenylmethylsulfonyl fluoride (Sigma-Aldrich, Missouri, USA). Samples were then vortexed and centrifuged at 4000×*g* for 40 min at 4 °C. The protein lysate was collected and kept at −80 °C until assayed.

### Quantification of cytokine production in plasma, EBC and PBMC cell lysates via ELISA

Cytokine expression was quantified in plasma (100 µl), which had undergone a two-fold dilution and undiluted EBC (100 µl). Protein concentrations in PBMC cell lysates were determined via the Bradford method (Bradford 1976). IFNγ protein levels were measured in 17 µg of total protein from PBMC cell lysates of patients and controls. IFNγ, IL-27 (R&D Systems, MN, USA) and IL-27p28 (BioScientific, GeneWorks, SA, Australia) levels were determined by specific ELISAs with detection limits of 15.6, 156.3 and 75 pg/ml, respectively. Assays were performed according to the manufacturer’s instructions. Absorbance was determined by spectrophotometry (SpectraMaxPlus Plate Reader, Molecular Devices, Surrey, UK) at manufacturer-specific wavelengths and the concentration in sample was determined by linear interpolation.

### Western blotting for T-bet

Equal amounts of PBMC cell lysate protein (4 µg) from both patient and healthy controls were loaded, subjected to 10 % SDS–polyacrylamide gel electrophoresis and were electrophoretically transferred to a polyvinylidene fluoride (PVDF) membrane (Millipore, Massachusetts, USA). Non-specific sites were blocked with 5 % BSA in TBST (Tris-buffered saline and 0.1 % Tween-20) for 1 h at room temperature. The membrane was washed five times for 5 min with PBST (phosphate-buffered saline and 0.05 % Tween-20) and incubated overnight at 4 °C with a polyclonal rabbit anti-human T-bet specific primary antibody (1:1000 final dilution) (Cell Signalling Technologies, MA, USA). After five washes with PBST, the membrane was incubated with goat anti-rabbit IgG HRP-conjugated secondary antibody (1:3000 final dilution, Cell Signalling Technologies). Protein bands were visualised with an enhanced chemiluminescence kit (PerkinElmer, MA, USA) using the ImageQuant LAS4000 (GE Healthcare Life Sciences, New Jersey, USA). Bands were analysed semi-quantitatively using ImageJ software (http://rsbweb.nih.gov/ij/) for T-bet quantification. To adjust for differences in image densities between membranes, the image densities of all bands were normalised to a standard sample which was repeated on all gels.

### Statistics

GraphPad Prism 6.0 software (La Jolla, CA, USA) was used for data analysis. The D’Agostino & Pearson omnibus normality test was used to test data for normality. Subject to data set normality, the data were analysed using unpaired *t* tests or Mann–Whitney *U* tests as appropriate. *p* < 0.05 was considered to be statistically significant. Data are expressed as mean ± SD or median ± range.

## Results

### Subject characteristics

Sarcoidosis patients (*n* = 18) and healthy controls (*n* = 21) were recruited (see Table 2 for full subject characteristics). Sarcoidosis patients had an elevated mean serum ACE level (48.9 U/L, NR < 42 U/L) and, compared to controls, demonstrated peripheral lymphopenia (*p* = 0.0004) typical of sarcoidosis (Iannuzzi and Fontana 2011).

**Table 2:** Clinical characteristics and demographics of sarcoidosis patients and healthy controls

Parameters	Healthy controls (*n* = 21)	Sarcoidosis patients (*n* = 18)
Male/female	7/14	11/7
Age (years)	39.7 ± 19.7	50.3 ± 10.5
Disease duration (years)	–	6.72 ± 6.71
Serum ACE (U/L) Normal <42 U/L		48.9 ± 19.8
Blood WCC (×10^6^ml)	4.38 ± 0.97	3.87 ± 1.26
Lymphocytes (%)*	32.9 ± 7.43	23.2 ± 6.98
Monocytes (%) median (min, max)	4.78 ± 1.82	6.43 ± 3.98
Neutrophils (%)*	59.05 ± 15.5	70.40 ± 8.33^b^
Pulmonary function		
FEV_1_ (% predicted)	96.55 ± 12.03	92.2 ± 13.6
FVC (% predicted)	92.9 ± 14.2	86.04 ± 26.7
FEV_1_/FVC ratio (% predicted)*	87.9 ± 7.0	96.5 ± 12.1
DLCO (% predicted)	–	70.5 ± 13.9
Radiological stage 0/I/II/III/IV^a^	–	2/2/8/0/6
Smoking history Non-smoker/ex-smoker/current smoker	15/6/0	10/7/1
Affected organs	–	
Lungs		16
Lymph nodes		1
Eyes		7
Kidneys		2
Others (spleen, liver, skin, vertebra, bone, brain)		12
Löfgren’s syndrome		2
Immunosuppressive treatment^c^ Yes/no	–	4/14
PBMC RNA concentration (µg/ml) Mean ± SD	380.1 ± 264.8	326.1 ± 120.5

Parametric variables are presented as mean ± SD. Differences between healthy controls and sarcoidosis patients (* *p* < 0.05). Values were based on unpaired *t* tests. *p* < 0.05 was considered significant

^a^Pulmonary radiological staging was scored according to the Scadding sarcoidosis chest X-ray staging system (Scadding 1961), Stage 0: no intra-thoracic involvement, Stage I: bilateral hilar lymphadenopathy, Stage II: bilateral hilar lymphadenopathy with parenchymal infiltration, Stage III: parenchymal infiltrates and Stage IV: lung fibrosis

^b^Probably a reflection of the percentage change due to the decrease in lymphocytes

^c^Patients were on one or a combination of the following immunosuppressive medications—prednisolone, azathioprine and mycophenolate

### T_H_1 cytokine mRNA expression in PBMCs

**Fig. 1:**
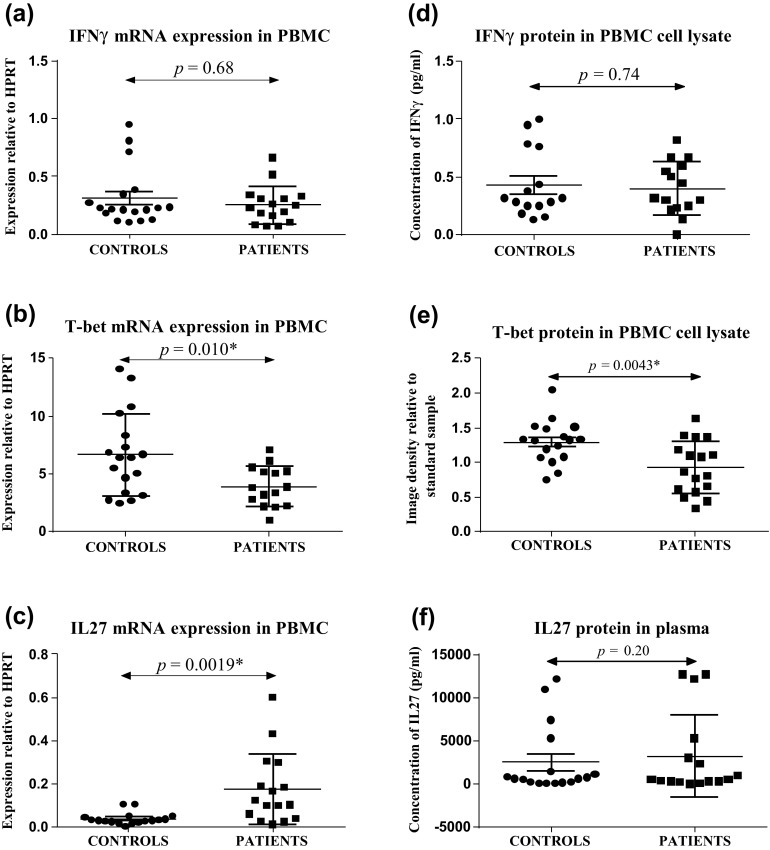
Expression of IFNγ, T-bet and IL-27 mRNA **a**–**c** in PBMC and protein **d**, **f** in plasma or PBMC cell lysates. Data represented as mean ± SD

There were no statistical differences in PBMC IFNγ mRNA levels between controls and sarcoidosis patients (*p* = 0.68, Fig. 1a). Compared to healthy controls, T-bet mRNA levels were lower in sarcoidosis patients (*p* = 0.010, Fig. 1b), while IL-27 mRNA levels were higher in sarcoidosis patients (*p* = 0.019, Fig. 1c).

To analyse the ability of T_H_1 cytokine mRNA levels to predict disease severity, sarcoidosis patients were further divided according to radiological stages. The mRNA expression levels of all cytokines did not vary significantly between the radiological stages (IFNγ: *p* = 0.63; T-bet: *p* = 0.10; IL-27: *p* = 0.086) (Online Resources 1 and 2). When patients were grouped according to treatment status, there were no significant differences between the groups for all T_H_1 cytokines studied (IFNγ: *p* = 0.71; T-bet: *p* = 0.31; IL-27: *p* = 0.26) (Online Resources 1 and 2).

### T_H_1 cytokine protein expression in plasma and/or PBMC cell lysate

**Fig. 2:**
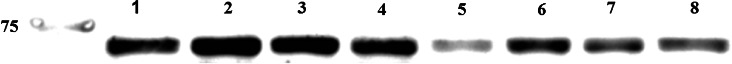
Western blot of T-bet protein. Western blot of T-bet in PBMC cell lysates of healthy controls (*lanes 1–4*) and patients (*lanes 5–8*). The sample in *lane 1* was used as a standard sample to normalise image densities within and between gels. T-bet was detected as a band of ~65 kDa

Comparative analysis demonstrated no significant differences in IFNγ in PBMC cell lysates or total IL-27 protein levels in the plasma of controls and sarcoidosis patients (IFNγ: *p* = 0.74 Fig. 1d; IL-27: *p* = 0.94 Fig. 1f). The more sensitive IL-27p28 assay showed six sarcoid subjects versus one control subject had detectable levels (mean 79 ± 110 vs. 29 ± 25 pg/ml, respectively, *p* = 0.026). T-bet protein was detected as bands of 65 kDa (Fig. 2). Densitometric analysis demonstrated that, compared to controls, T-bet protein expression in PBMCs was lower in sarcoidosis patients (*p* = 0.0043, Fig. 1e).

When analysed according to radiological stage and treatment status, there were no significant differences between controls and sarcoidosis patients for all T_H_1 cytokines (radiological staging: IFNγ: *p* = 0.59, T-bet: *p* = 0.14, IL-27: *p* = 0.85; treatment status: IFNγ: *p* = 0.43, T-bet: *p* = 0.27, IL-27: *p* = 0.97) (Online Resource 1 and 2).

### IL-27 expression in EBC

Samples that had undetectable IL-27 were arbitrary assigned a value equivalent to half of the lowest level detected by the kit (Edmé et al. 2008). IL-27 was detected in 6/20 controls and 6/18 sarcoidosis patients. The mean IL-27 concentrations were 3.1 ± 4.2 pg/ml (mean ± SD) in controls and 1.8 ± 3.0 pg/ml in sarcoidosis patients. Nonetheless, this difference was not statistically significant (*p* = 0.57) (Online Resource 3).

## Discussion

This study aimed to quantify the expression of T_H_1 cytokines in the peripheral blood of sarcoidosis patients (Loke et al. 2013), but because post-transcriptional regulation has been known to make mRNA levels poor approximations for protein levels (Hack 2004), both mRNA and protein levels of T_H_1 cytokines in the peripheral blood of sarcoidosis patients were studied.

T-bet has been shown to orchestrate T_H_1 responses especially in the transcription of IFNγ (Djuretic et al. 2007). In comparison with healthy controls, T-bet mRNA and protein expression were lower in the sarcoidosis patients in keeping with our hypothesis, suggesting T-bet involvement in the immunopathogenesis of sarcoidosis. To date, only Kriegova et al. (2011) have examined the role of T-bet in sarcoidosis and demonstrated that T-bet mRNA expression levels were increased in BAL lymphocytes. While they did not examine T-bet protein expression levels, one could surmise that T-bet expression levels in the periphery are decreased at these sites of anergy but increased at sites of disease suggesting T-bet involvement in the peripheral anergy underpinning sarcoidosis. This inference is further reinforced by our findings which showed consistency between mRNA and protein expression levels and other findings of Kriegova et al. (in the same study), where T-bet mRNA levels were found to correlate with IFNγ and other chemokines associated with T_H_1 immunity, reinforcing its role as a key modulator of T_H_1 immunity.

This study is the first to demonstrate increased PBMC IL-27 mRNA expression in sarcoidosis patients compared to healthy controls. There was a significant trend to elevated IL27p28 levels in the plasma from sarcoidosis patients but not in the IL-27 assay, suggesting that the levels are low and very sensitive assays are required. The low levels could be attributed to the short in vivo half-life of IL-27 (Do et al. 2012; Pot et al. 2010) or differences in the half-life of IL-27 between patients and controls, and possibly translational repression.

There were no significant differences between PBMC IFNγ mRNA expression levels in sarcoidosis patients and controls in keeping with previous findings (Swider et al. 1995). Intracellular PBMC IFNγ protein expression levels were measured using ELISA in PBMC cell lysates. To our knowledge, this was the first time this experimental approach was used to quantify intracellular IFNγ protein in sarcoidosis patients. Mirroring mRNA data and data from another study that analysed intracellular IFNγ protein expression using flow cytometry (Hill et al. 2008), IFNγ protein levels were not elevated in PBMCs of sarcoidosis patients, suggesting that mechanisms within PBMCs may be implicated for the suppression of IFNγ. Small, 20–25 nucleotide, non-coding RNAs, known as microRNAs (miRNAs) have been shown to be natural repressors of gene translation (Jung et al. 2010). Aberrant miRNA expressions have been observed in sarcoidosis patients (Crouser et al. 2012). Additionally, members of the miR-29 family have been known to target IFNγ (Ma et al. 2011) and T-bet (Steiner et al. 2011) thereby influencing T_H_1 immunity. The low levels of T-bet mRNA and protein observed in this study suggest that miR-29a could have a role in modulating the T_H_1 profile within PBMCs by suppressing IFNγ and T-bet.

This is the first study to detect IL-27 in EBC of sarcoidosis patients. EBC IL-27 levels were not significantly different between sarcoidosis controls and patients. Although one may conclude that IL-27 may not be implicated in the immunopathogenesis of pulmonary sarcoidosis, all 6/18 sarcoidosis patients who had detectable IL-27 EBC levels had pulmonary sarcoidosis. In view of the low IL-27 levels, this observation begets further investigation using higher sensitivity assays.

To assess the ability of the aforementioned immunomodulators to delineate clinical phenotype and assess their expression levels in relation to treatment status, we analysed immunomodulator expression according to the radiological stage and treatment status of sarcoidosis patients. In these small subgroups, radiological staging and immunosuppressive treatment was not associated with the expression of these modulators in the peripheral blood and EBC. The expression of these modulators is not influenced by radiological staging or immunosuppression in this study, but these findings are limited by small residual sample sizes indicating the need to confirm these results in a larger cohort of sarcoidosis patients.

## Conclusion

This study is the first to document depressed T-bet mRNA and protein expression in the peripheral blood of sarcoidosis patients compared to controls, suggesting a role for T-bet in modulating the T_H_1 immunological response and a possible role of T-bet in the sarcoidosis peripheral anergy. We also documented elevated IL-27 mRNA and IL-27p28 levels in PBMCs of sarcoidosis patients compared to controls. Given that this same trend of IL-27 expression was not observed in IL-27 protein levels in the plasma and that there were no differences in EBC IL-27 protein expression between patients and controls, IL-27 may have limited involvement in sarcoidosis. Larger scale studies are required to validate the reproducibility of these findings.

## Electronic supplementary material

Supplementary material 1 (DOCX 472 kb)
