# Supplementary material for: T-bet and interleukin-27: possible TH1 immunomodulators of sarcoidosis
Source: Inflammopharmacology. 2015 Aug 9;23(5):283–90. doi: 10.1007/s10787-015-0247-y (PMC4568011; doi:10.1007/s10787-015-0247-y)
Supplement: Supplementary file 1 — Supplementary material 1 (DOCX 472 kb) [file 10787_2015_247_MOESM1_ESM.docx]

**Title:** T-bet and interleukin-27: possible T_H_1 immunomodulators of sarcoidosis

**Article type:** Original article

**Corresponding author:** Wei Sheng Joshua Loke, MB BS VI

**First Author:** Wei Sheng Joshua Loke, MB BS VI

**Order of authors:** Joshua Loke^1,2^, Araluen Freeman^1^, Linda Garthwaite^1^, Silvie Prazakova^1,2^, Mijeong Park^1^, Kenneth Hsu^1^, Paul S. Thomas^1,2^, and Cristan Herbert^1^

**Author affiliations/location:**

1. Inflammation and Infection Research Centre, School of Medical Sciences, UNSW, 2052, Australia.

2. Department of Respiratory Medicine, Prince of Wales Hospital, Randwick, NSW 2031, Australia.

**Corresponding author:**

Wei Sheng Joshua Loke, Department of Respiratory Medicine, Prince of Wales Hospital, Randwick, NSW 2031, Australia. Inflammation and Infection Research Centre, UNSW Australia, Sydney, 2052, Australia. Email: [z3301409@student.unsw.edu.au](mailto:z3301409@student.unsw.edu.au) ([joshua_loke_w_s@hotmail.com](mailto:joshua_loke_w_s@hotmail.com)); Telephone: +61 2 9382 4620; Fax: +61 2 9382 4627

**Email address of other authors:**

Araluen Freeman [araluen.freeman@unsw.edu.au](mailto:araluen.freeman@unsw.edu.au)

Linda Garthwaite [l.garthwaite@unsw.edu.au](mailto:l.garthwaite@unsw.edu.au)

Silvie Prazakova [s.prazakova@unsw.edu.au](mailto:s.prazakova@unsw.edu.au)

Mijeong Park [mijeong.park@unsw.edu.au](mailto:mijeong.park@unsw.edu.au)

Kenneth Hsu [k.hsu@unsw.edu.au](mailto:k.hsu@unsw.edu.au)

Cristan Herbert [c.herbert@unsw.edu.au](mailto:c.herbert@unsw.edu.au)

**Online resource**

Online resource 1: Expression of IFNγ, T-bet and IL-27 mRNA (a-c) in PBMC, and protein (d-f) in plasma or PBMC cell lysates of sarcoidosis patients according to radiological stage. Data represented as mean ± SD.

Online resource 2: Expression of IFNγ, T-bet and IL-27 mRNA (a-c) in PBMC, and in protein (d-f) in plasma or PBMC cell lysates of sarcoidosis patients according to treatment status. Data represented as mean ± SD.

Online resource 3: Comparison of EBC IL-27 concentrations in healthy controls and sarcoidosis patients. Data represented as mean ± SD.
